# Increase in Hepatitis A Virus Infections — United States, 2013–2018

**DOI:** 10.15585/mmwr.mm6818a2

**Published:** 2019-05-10

**Authors:** Monique A. Foster, Megan G. Hofmeister, Benjamin A. Kupronis, Yulin Lin, Guo-Liang Xia, Shaoman Yin, Eyasu Teshale

**Affiliations:** 1 Division of Viral Hepatitis, National Center for HIV, Viral Hepatitis, STD, and TB Prevention, CDC.

Hepatitis A virus (HAV) is primarily transmitted fecal-orally after close contact with an infected person (*1*); it is the most common cause of viral hepatitis worldwide, typically causing acute and self-limited symptoms, although rarely liver failure and death can occur (*1*). Rates of hepatitis A had declined by approximately 95% during 1996–2011; however, during 2016–2018, CDC received approximately 15,000 reports of HAV infections from U.S. states and territories, indicating a recent increase in transmission (*2*,*3*). Since 2017, the vast majority of these reports were related to multiple outbreaks of infections among persons reporting drug use or homelessness (*4*). In addition, increases of HAV infections have also occurred among men who have sex with men (MSM) and, to a much lesser degree, in association with consumption of imported HAV-contaminated food (*5*,*6*). Overall, reports of hepatitis A cases increased 294% during 2016–2018 compared with 2013–2015. During 2016–2018, CDC tested 4,282 specimens, of which 3,877 (91%) had detectable HAV RNA; 565 (15%), 3,255 (84%), and 57 (<1%) of these specimens were genotype IA, IB, or IIIA, respectively. Adherence to the Advisory Committee on Immunization Practices (ACIP) recommendations to vaccinate populations at risk can help control the current increases and prevent future outbreaks of hepatitis A in the United States (*7*).

Hepatitis A infections among persons who meet the Council of State and Territorial Epidemiologists (CSTE) hepatitis A case definition (https://wwwn.cdc.gov/nndss/conditions/hepatitis-a-acute/) are notified to CDC through the National Notifiable Diseases Surveillance System (NNDSS). Cases reported to CDC through NNDSS during 2013–2018 were used to calculate percent change (2013–2015 versus 2016–2018) by state and mapped using RStudio software (version 1.2.1335; RStudio, Inc.). Serum specimens from CSTE confirmed cases submitted to the CDC laboratory were tested for HAV RNA by polymerase chain reaction, and isolated virus was amplified to characterize a 315–base-pair fragment of the VP1/P2B region, which defines the genotype of the virus.

Overall, reports of hepatitis A cases increased 294% during 2016–2018 compared with 2013–2015 (Figure). Eighteen states had lower case counts during 2016–2018 compared with 2013–2015. Nine states and Washington, DC had an increase of approximately 500%. During 2013–2018, 4,508 HAV anti-immunoglobulin M–positive specimens underwent additional testing at CDC. During 2013–2015, 226 specimens underwent additional testing, of which 197 (87%) had detectable HAV RNA; of the RNA-positive specimens, 76 (39%), 121 (61%), and 0 (0%) tested positive for a genotype IA, IB, or IIIA viral strain, respectively. In comparison, 4,282 specimens were tested by CDC during 2016–2018, of which 3,877 (91%) had detectable HAV RNA; 565 (15%), 3,255 (84%), and 57 (<1%) of these specimens were genotype IA, IB, or IIIA, respectively.

**FIGURE:**
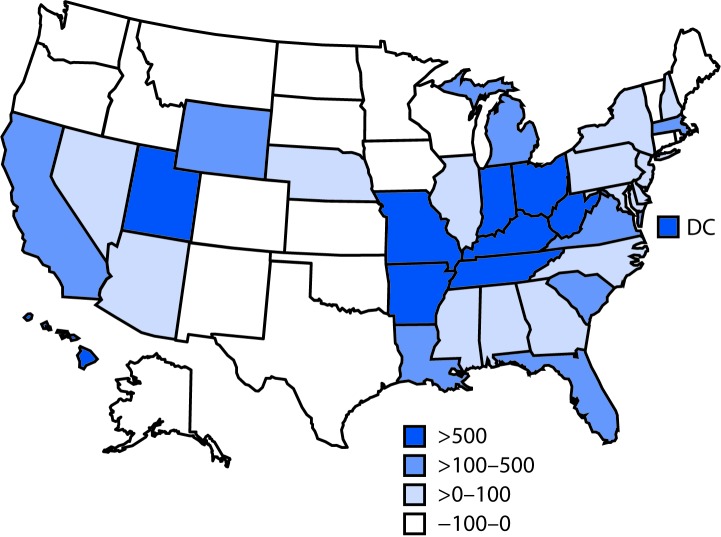
Percent change in reported hepatitis A infections, by state — National Notifiable Diseases Surveillance System, United States, 2013–2015 and 2016–2018* **Abbreviation:** DC = District of Columbia. * 2017 and 2018 case counts are provisional.

## Discussion

The number of hepatitis A infections reported to CDC increased during 2016–2018, along with the number of specimens from infected persons submitted to CDC for additional testing. In the past, outbreaks of hepatitis A virus infections occurred every 10–15 years and were associated with asymptomatic children (*8*). With the widespread adoption of universal childhood vaccination recommendations (https://www.cdc.gov/mmwr/preview/mmwrhtml/rr5507a1.htm), asymptomatic children are no longer the main drivers of hepatitis A outbreaks (*3*,*9*). Although the overall incidence rate of HAV infections has decreased within all age groups, a large population of susceptible, unvaccinated adults who were not infected by being exposed to the virus during childhood remain vulnerable to infection by contaminated foods (typically imported from countries with endemic HAV transmission) and recently, on a much larger scale, through behaviors that increase risk for infection in certain vulnerable populations, such as drug use (*3*).

Increasingly, molecular epidemiology is employed by public health laboratories to better characterize hepatitis A transmission patterns. When combined with reliable epidemiologic data, these laboratory data can be used to identify transmission networks and confirm the source of exposure during common-source outbreaks, facilitating prompt and effective public health response. Historically, genotype IA has been the most common genotype circulating in North and South America. During 2013–2018, HAV genotype IB predominated in the United States. Increasing numbers of genotype IIIA were seen, a genotype that is considered rare in the United States.

Decreasing new infections from hepatitis A virus can be achieved and sustained by maintaining a high level of population immunity through vaccination. There is no universal vaccination recommendation for adults in the United States; however, ACIP does recommend vaccination for adults who plan travel to HAV-endemic countries, MSM, persons who use drugs, persons with chronic liver disease, and recently, persons experiencing homelessness (*7*). Continued efforts to increase hepatitis A vaccination coverage among the ACIP-recommended risk groups is vital to halting the current hepatitis A outbreaks and reducing overall hepatitis A incidence in the United States.

**Summary**

**What is already known about this topic?**

Hepatitis A is a vaccine-preventable viral infection of the liver that is primarily transmitted through consumption of microscopic amounts of feces.

**What is added by this report?**

During 2016–2018, reports of hepatitis A infections in the United States increased by 294% compared with 2013–2015, related to outbreaks associated with contaminated food items, among men who have sex with men, and primarily, among persons who report drug use or homelessness.

**What are the implications for public health practice?**

Increasing vaccination among groups at risk for hepatitis A infection might halt ongoing outbreaks and prevent future outbreaks.

## References

[1] Franco E, Meleleo C, Serino L, Sorbara D, Zaratti L. Hepatitis A: epidemiology and prevention in developing countries. World J Hepatol 2012;4:68–73. 10.4254/wjh.v4.i3.68 22489258 PMC3321492

[2] CDC. Viral hepatitis surveillance, United States 2016. Atlanta, GA: US Department of Health and Human Services, CDC; 2017. https://www.cdc.gov/hepatitis/statistics/2016surveillance/pdfs/2016HepSurveillanceRpt.pdf

[3] CDC. National notifiable infectious diseases: weekly tables, 2018. Atlanta, GA: US Department of Health and Human Services, CDC; 2018. https://wonder.cdc.gov/nndss/static/2018/52/2018-52-table2H-H.pdf

[4] Foster M, Ramachandran S, Myatt K, Hepatitis A virus outbreaks associated with drug use and homelessness—California, Kentucky, Michigan, and Utah, 2017. MMWR Morb Mortal Wkly Rep 2018;67:1208–10. 10.15585/mmwr.mm6743a3 30383739 PMC6319801

[5] Viray MA, Hofmeister MG, Johnston DI, Public health investigation and response to a hepatitis A outbreak from imported scallops consumed raw—Hawaii, 2016. Epidemiol Infect 2018;147:1–8. 30326986 10.1017/S0950268818002844

[6] Latash J, Dorsinville M, Del Rosso P, Notes from the field: increase in reported hepatitis A infections among men who have sex with men—New York City, January–August 2017. MMWR Morb Mortal Wkly Rep 2017;66:999–1000. 10.15585/mmwr.mm6637a7 28934181 PMC5657783

[7] Doshani M, Weng M, Moore KL, Romero JR, Nelson NP. Recommendations of the Advisory Committee on Immunization Practices for use of hepatitis A vaccine for persons experiencing homelessness. MMWR Morb Mortal Wkly Rep 2019;68:153–6. 10.15585/mmwr.mm6806a6 30763295 PMC6375653

[8] Murphy TV, Denniston MM, Hill HA, Progress toward eliminating hepatitis A disease in the United States. MMWR Suppl 2016;65:29–41. 10.15585/mmwr.su6501a6 26916458

[9] Klevens RM, Denniston MM, Jiles-Chapman RB, Murphy TV. Decreasing immunity to hepatitis A virus infection among US adults: findings from the National Health and Nutrition Examination Survey (NHANES), 1999–2012. Vaccine 2015;33:6192–8. 10.1016/j.vaccine.2015.10.009 26476364

